# Adaptive Deep Brain Stimulation for Parkinson's Disease: Navigating the Roadblocks to Clinical Implementation

**DOI:** 10.1002/mds.70284

**Published:** 2026-04-01

**Authors:** Thomas Koeglsperger, Maximilian Scherer, Andrea A. Kühn, Alexandre Eusebio, Alfonso Fasano, Joachim K. Krauss

**Affiliations:** ^1^ German Center for Neurodegenerative Diseases (DZNE) Munich Germany; ^2^ Department of Neurology LMU University Hospital, LMU Munich Munich Germany; ^3^ Berlin Institute of Health at Charité – Universitätsmedizin Berlin Berlin Germany; ^4^ Bernstein Center for Computational Neuroscience Humboldt Universität zu Berlin Berlin Germany; ^5^ Exzellenzcluster NeuroCure Charité ‐ Universitätsmedizin Berlin Berlin Germany; ^6^ Berlin School of Mind and Brain Humboldt ‐ Universität zu Berlin Berlin Germany; ^7^ German Center for Neurodegenerative Diseases (DZNE) Berlin Germany; ^8^ APHM, Department of Neurology and Movement Disorders Timone University Hospital Marseille France; ^9^ Aix‐ Marseille University Institut de Neurosciences de la Timone, UMR 7289 CNRS‐AMU Marseille France; ^10^ Edmond J Safra Program in Parkinson's disease and Morton and Gloria Shulman Movement Disorders Centre, Toronto Western Hospital, UHN and Division of Neurology University of Toronto Toronto Ontario Canada; ^11^ Krembil Brain Institute Toronto Ontario Canada; ^12^ Department of Biomedical Sciences Humanitas University Milan Italy; ^13^ IRCCS Humanitas Research Hospital Milan Italy; ^14^ Department of Neurosurgery Hannover Medical School Hannover Germany; ^15^ Center for Systems Neuroscience Hannover Germany

**Keywords:** adaptive DBS, biomarker, control algorithm, deep brain stimulation (DBS), Parkinson's disease

## Abstract

Adaptive deep brain stimulation (aDBS) represents an important evolution in the treatment of Parkinson's disease (PD), building on conventional DBS (cDBS) by adjusting stimulation in response to real‐time physiological signals. By enabling dynamic targeting of disease‐related neural activity, aDBS offers the potential for more precise modulation of motor symptoms. Additional anticipated advantages include reduced stimulation‐related side effects and improved energy efficiency, supporting long‐term device performance. Although clinical uptake is still at an early stage, growing experience has highlighted both opportunities and areas requiring further refinement. Key challenges include inter‐individual variability in biomarker expression, diversity in programming approaches, and ongoing debate regarding optimal thresholds and response latencies. The clinical significance of short‐term local field potential (LFP) recordings continues to be actively investigated, particularly in the context of signal artifacts, physiological variability, and current hardware limitations. Beyond technical considerations, factors such as patient selection, ethical frameworks, and cost‐effectiveness remain important determinants of broader implementation. Continued progress will depend on the development of robust and flexible control strategies that incorporate multimodal biomarkers, including wearable‐derived motor metrics and patient‐reported outcomes, to support personalized therapy. With an expanding evidence base and recent regulatory approvals, aDBS is increasingly transitioning from an experimental concept to a viable clinical tool. Future efforts should prioritize the translation of research paradigms into scalable clinical workflows that effectively balance automation with individualized patient care. © 2026 The Author(s). *Movement Disorders* published by Wiley Periodicals LLC on behalf of International Parkinson and Movement Disorder Society.

Deep brain stimulation (DBS) of the subthalamic nucleus (STN) or globus pallidus internus (GPi) is an established therapy for advanced Parkinson's disease (PD), providing sustained motor improvement, reduced medication burden, and improved quality of life.^1^, ^2^, ^3^ Clinical outcomes depend strongly on postoperative programming, a complex and iterative process to balance efficacy and side effects.^4^, ^5^, ^6^, ^7^ Conventional DBS (cDBS) delivers continuous stimulation independent of clinical or electrophysiological state and, although effective, may be associated with stimulation‐induced side effects^8^, ^9^, ^10^, ^11^, ^12^ and incomplete motor control.^13^ Adaptive DBS (aDBS) introduces closed‐loop control using real‐time physiological feedback to optimize stimulation delivery (Fig. 1; Table 1).

**FIG. 1:**
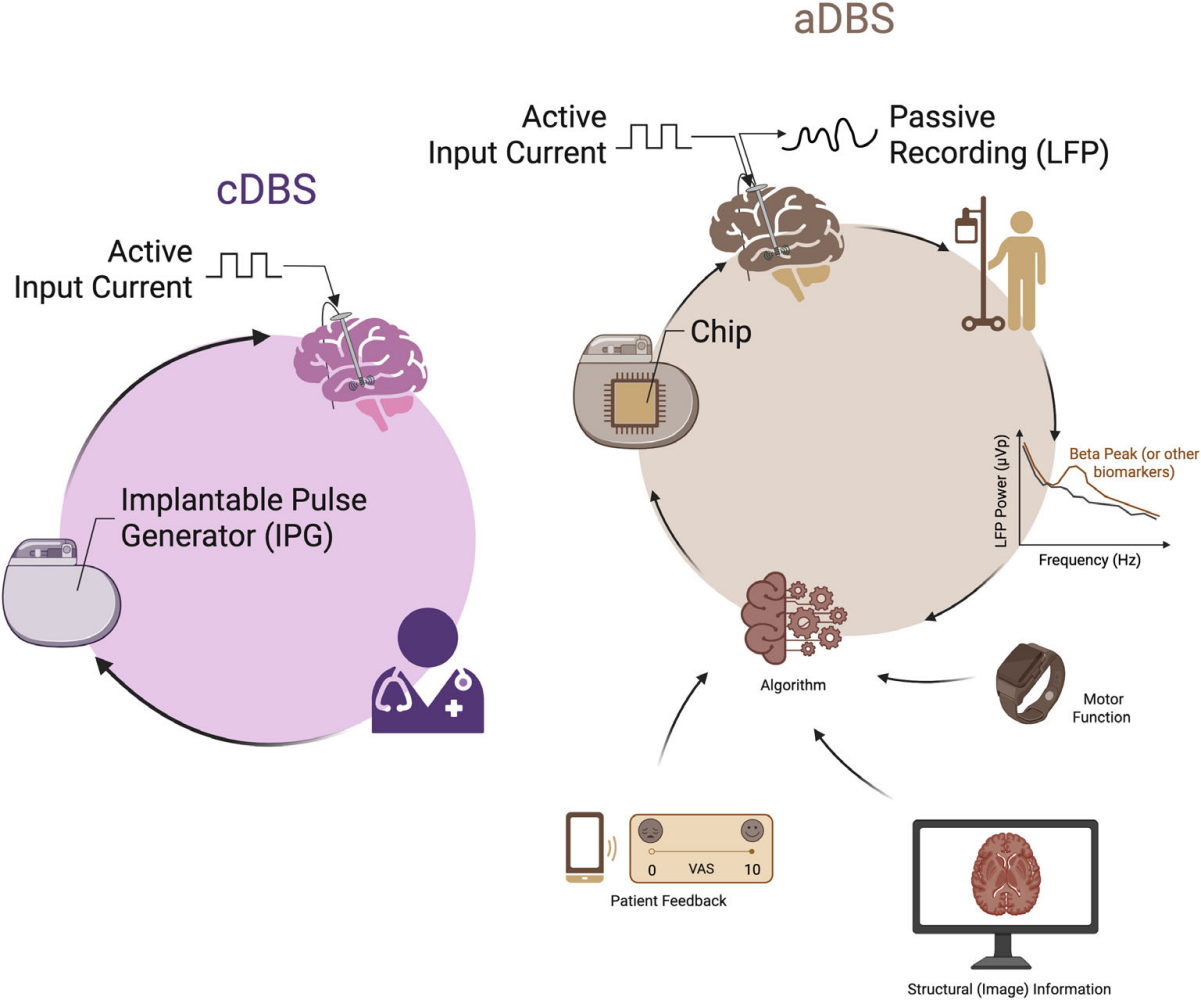
Schematic illustrating the integration of multiple feedback signals into adaptive deep brain stimulation (aDBS) control routine. [Color figure can be viewed at wileyonlinelibrary.com]

**TABLE 1:** . *Comparison of cDBS and aDBS*

Feature	cDBS	aDBS	Key implication
Stimulation pattern	Continuous, open‐loop	Feedback‐controlled, closed‐loop	aDBS tailors therapy to patient state
Biomarker use	None	β, γ, PAC, wearable signals	aDBS requires reliable biomarkers
Adjustability	Manual programming	Automated adaptation	Reduces need for clinician intervention
Energy use	Higher overall	Lower stimulation but added sensing power	Battery savings uncertain, may depend on system
Clinical evidence	Large, long‐term	Emerging, limited cohorts	aDBS still in translational phase
Best candidate profile	Broad PD population	Patients with stable biomarker expression	Patient selection remains critical

*Note*: Key distinguishing features of stimulation patterns, biomarker utilization, adaptability, energy demands, level of clinical evidence, and patient selection considerations are summarized. The table highlights how aDBS introduces feedback‐based modulation, but requires reliable physiological biomarkers and individualized control strategies.

Abbreviations: cDBS, conventional DBS; aDBS, adaptive DBS; PAC, phase‐amplitude coupling; PD, Parkinson's disease.

Current aDBS paradigms primarily use LFPs, especially β‐band oscillations (13–30 Hz), as biomarkers of motor state, although alternative signals and control strategies are under investigation.^14^ By adjusting stimulation in real time, aDBS aims to deliver more targeted neuromodulation. The Medtronic Percept PC (Medtronic, Minneapollis, MN, USA), introduced in 2020, was the first clinically approved DBS system enabling chronic sensing outside epilepsy,^15^ and additional platforms from AlphaDBS (Newronica, Milan, Italy), PINS Medical (Pins Medical Co., Beijing, China), and Picostim (Amber Therapeutics, Didcot, GB) continue to expand aDBS capabilities and biomarker validation.^16^, ^17^, ^18^ Although early studies suggest improved energy efficiency and fewer side effects, clear superiority over cDBS has not been established,^19^ although recent data indicate benefit in selected patients.^20^, ^21^ While research is also extending to other movement and psychiatric disorders,^22^, ^23^, ^24^, ^25^, ^26^, ^27^, ^28^ we focus here on key gaps and controversies in aDBS for PD, the most common DBS indication.

## What We Know

### Control Strategies

**FIG. 2:**
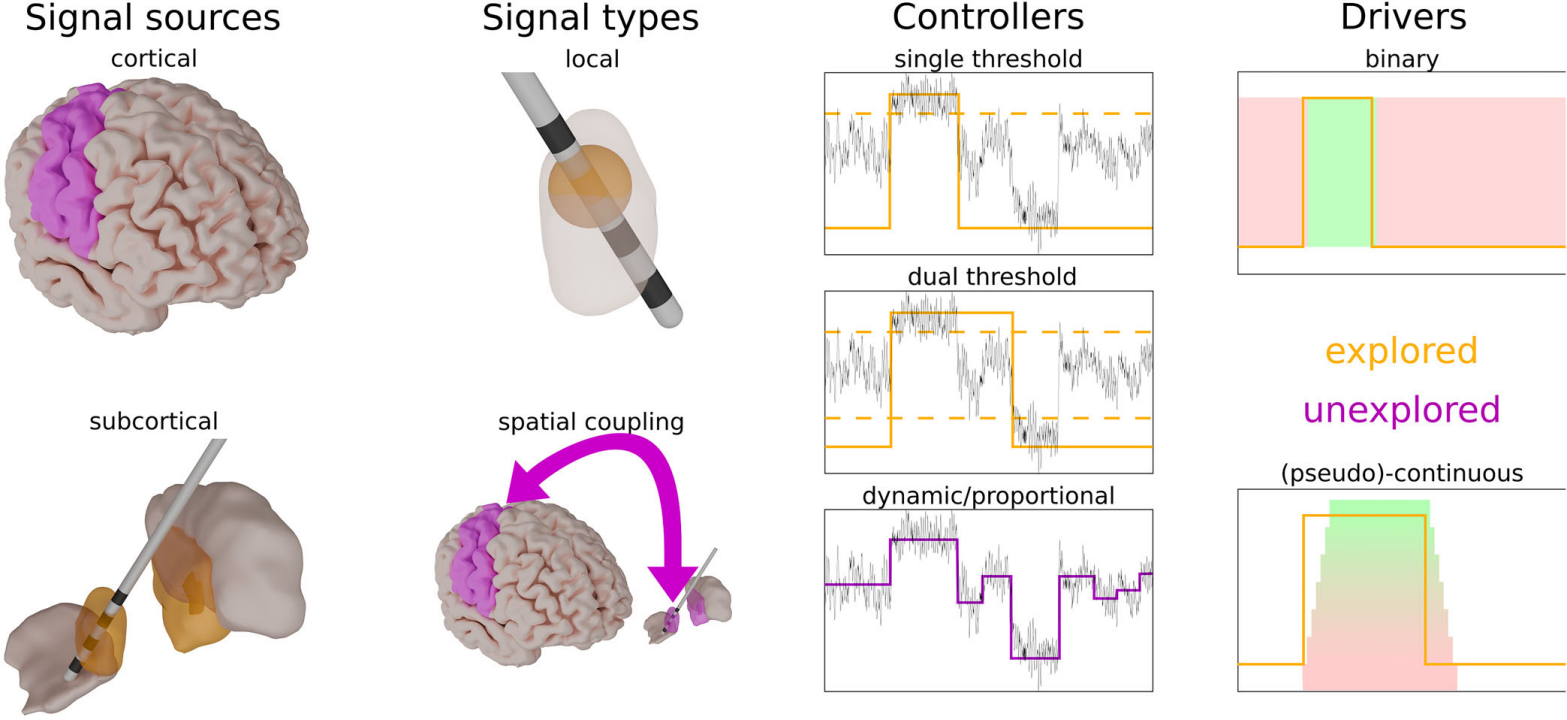
Schematic overview over feedback signals, sources, controller types, and drivers. [Color figure can be viewed at wileyonlinelibrary.com]

Current aDBS systems translate electrophysiological biomarkers into real‐time stimulation via a controller–driver architecture. Controllers are typically single‐threshold (ST), or dual‐threshold (DT) (Fig. 2).^18^, ^29^, ^30^ ST controllers activate stimulation when a biomarker crosses a preset threshold,^31^, ^32^, ^33^, ^34^ whereas DT controllers use upper and lower thresholds to buffer fluctuations.^35^ Drivers implement either binary or (pseudo‐)continuous stimulation. ST approaches rapidly suppress pathological β bursts (13–30 Hz),^36^, ^37^, ^38^, ^39^, ^40^ whereas DT approaches track broader oscillatory dynamics, including β suppression or γ activity (60–90 Hz) associated with effective motor performance.^41^, ^42^, ^43^

### Biomarkers

Most PD devices analyze neural oscillations via onboard Fourier transforms focusing on single β peaks (Table 2). In PD, oscillatory activity across frequency bands relates to distinct motor (tremor, bradykinesia, and freezing) and non‐motor symptoms.^15^, ^44^, ^45^, ^46^, ^47^, ^48^, ^49^, ^50^, ^51^, ^52^, ^53^ β‐band oscillations in the STN and GPi are a hallmark of bradykinesia and (to a lesser extent) rigidity, and are exaggerated *off* medication, and align spatially with optimal dorsolateral STN stimulation sites.^54^, ^55^, ^56^, ^57^, ^58^ Suppression of elevated β activity coincides with symptom relief and correlates with disease severity, establishing β power as a real‐time aDBS biomarker.^59^, ^60^, ^61^, ^62^, ^63^ Subthalamic high‐frequency oscillations (HFOs) (~200–500 Hz) are pro‐kinetic biomarkers reduced in untreated PD and shifted to approximately 350 Hz with dopaminergic therapy,^64^, ^65^, ^66^ but are difficult to capture with current aDBS hardware because of sampling limitations. In contrast, finely tuned gamma (FTG) (60–70 Hz) lies within the detectable range of implanted systems, shows motor‐related modulation, and can entrain to DBS frequency, making it a practical target for chronic aDBS despite being less studied than β oscillations.^51^, ^67^ DBS‐induced neural entrainment provides an additional candidate biomarker, with STN γ synchronizing to stimulation harmonics under dopaminergic therapy and correlating with improved motor function.^68^, ^69^, ^70^, ^71^, ^72^ Other advanced biomarkers include phase‐amplitude coupling (PAC) between β and HFOs, capturing interactions across frequency bands.^73^, ^74^, ^75^ Although conceptually promising, current hardware does not support on‐board PAC analysis. Beyond electrophysiology, non‐electrophysiological biomarkers such as wearable motion sensors (already incorporated into Medtronic's Percept device),^76^, ^77^, ^78^, ^79^, ^80^, ^81^ radiowave‐based systems combined with artificial intelligence (AI) analysis or motor function^82^, ^83^ and facial expressions, or voice^84^, ^85^ can provide high‐resolution motor state detection, potentially enabling real‐time aDBS control.

**TABLE 2:** Electrophysiological and peripheral biomarkers investigated for aDBS control in Parkinson's disease

Biomarker	Frequency/type	Clinical association	Strengths	Limitations	Current aDBS readiness/notes	Validation status
β LFP power	13–30 Hz	Rigidity, bradykinesia	Well‐validated, real‐time sensing; widely used	Absent in a proportion of patients; weak for tremor	Primary biomarker for aDBS	Validated in multiple cohorts (chronic human data)
β burst duration	13–30 Hz (time‐domain)	Symptom fluctuations	Captures dynamics better than power	Variable across patients	Increasing adoption; useful for feedback control	Single‐center/limited cohort (acute + early chronic)
TF	~4–6 Hz (resting tremor)	Tremor onset / intensity	Directly reflects peripheral tremor; may complement β	Latency of appearance must be considered; variable	Can be combined with β for tremor‐dominant PD	Single‐center/limited cohort
FTG	60–90 Hz	Medication state/dyskinesias	Reflects dopaminergic state; movement‐related	Less studied; lower SNR	Promising, emerging for levodopa‐dependent modulation	Single‐center feasibility (chronic human)
Stimulation‐entrained γ	~1/2 DBS frequency	Predicts motor fluctuations	Works even when β is suppressed by DBS	Requires more computation	Strong emerging candidate; real‐time implementation feasible	Single‐center feasibility (chronic human)
PAC (β–HFO coupling)	Cross‐frequency	Symptom severity; mechanistic insight	Provides mechanistic understanding	Not supported in current implants	Research phase; not yet clinically implemented	Experimental/offline only
HFO	200–500 Hz (slow HFO 200–300, fast HFO 300–500)	Movement onset, dopaminergic state	Fast changes allow rapid triggering; prokinetic	Less studied; requires high sampling rates	Potential for faster aDBS triggering, especially combined with TF and β	Intraoperative/short‐term only
Wearable kinematic sensors	n/a	Tremor, gait, OFF‐time, freezing of gait	High resolution; multi‐symptom monitoring	Requires integration with implant system	High potential for multimodal aDBS, especially gait and FOG	Validated in multiple cohorts (non‐invasive)
Patient reported feedback	n/a	Symptom perception, OFF periods, side effects	Captures subjective experience; complements objective signals	Subjective, low temporal resolution, compliance‐dependent	Can be integrated via apps or controllers to modulate aDBS in real‐time	Validated as outcome measure; exploratory as control signal

*Note*: Summary of principal candidate biomarkers, including associated frequency ranges, motor symptom correlates, strengths, limitations, and estimated readiness for clinical deployment. Note that validation refers to reproducibility of the biomarker–symptom relationship rather than clinical endorsement or demonstrated superiority in adaptive DBS trials.

Abbreviations: aDBS, adaptive deep brain stimulation; FOG, freezing of gait; FTG, finely tuned gamma; HFO, high‐frequency oscillations; LFP, local field potential; PAC, phase‐amplitude coupling; PD, Parkinson's disease; TF, tremor frequency.

### Signal Artifacts and Handling

Electrophysiological biomarkers show marked interindividual and intra‐individual variability influenced by movement, medication, and sleep.^86^, ^87^ Reliable use requires managing artifacts from physiological sources, stimulation, and external interference, which can alias into clinically relevant bands and trigger aDBS.^88^, ^89^, ^90^, ^91^ Artifact occurrence depends on system‐level interactions, complicating prediction. Although manual review can address multiple artifact types, current embedded aDBS safeguards are limited to basic amplitude limits, smoothing, and refractory periods,^32^, ^86^, ^88^, ^92^ which stabilize signals, but insufficiently mitigate movement‐related or external artifacts. For clinicians, the practical implication is that physiological artefacts—most notably electrocardiography‐related signals and movement‐induced artefacts—can contaminate β‐band recordings and may spuriously trigger or suppress adaptive stimulation algorithms if not adequately accounted for.

### Clinical Application

Because commercially available aDBS has only recently become accessible, long‐term outcome data remain limited, and most evidence derives from short‐term or intraoperative studies. Several investigations reported superior motor control with aDBS compared with cDBS,^18^, ^31^, ^32^ whereas others found comparable efficacy.^30^, ^33^, ^78^ A meta‐analysis of 19 small studies (all n < 15) suggested that aDBS may improve motor symptoms by approximately 33.9% while reducing stimulation energy by approximately 55% relative to cDBS,^19^ indicating potential efficiency benefits, although generalizability remains limited. A first real‐world application of the commercially available aDBS system showed a significant improvement in subjective well‐being and motor function with DT‐aDBS as compared to cDBS in a small cohort using EMA (European Medicines Agency) questionnaires.^20^, ^93^ The first large‐scale, home‐based evaluation of aDBS implemented with a commercial device is the ADAPT‐PD trial.^21^, ^94^, ^95^, ^96^ Sixty‐eight participants with established STN or GPi cDBS underwent baseline assessment, aDBS calibration, n = 57 met recruitment criteria, randomized single‐blind crossover testing of ST and DT modes (n = 30), and optional approximately 10‐month follow‐up (n = 40). Mechanistically, ADAPT‐PD offered ST‐controlled rapid, threshold‐based suppression of brief β bursts (>500 ms) common in low‐medication states^38^ with bilateral DBS adjustments and DT‐aDBS with a pseudo‐continuous control strategy with slower update intervals (2.5–5 minutes) tracking β fluctuations tied to levodopa pharmacodynamics, allowing independent hemispheric modulation,^96^ but with slower responsiveness.^35^

The revised primary endpoint—a maximum decrease of ON‐time without troublesome dyskinesias of at most 1 standard deviation compared to cDBS—was achieved in 91% of DT‐aDBS and 79% of ST‐aDBS participants. On average, aDBS, especially DT mode, increased ON‐time without troublesome dyskinesias by approximately 1.4 ± 3 hours relative to cDBS on the group level. On an individual level, ST mode yielded better outcomes in 13 patients and worse outcomes in 16, whereas DT mode was better in 22 and worse in 13. Seventeen of 27 participants preferred DT mode. Nonetheless, patients were nearly as likely to select an aDBS mode with inferior as with superior ON‐time performance. Although clinically meaningful for some patients, the approximately 1‐hour increase in daily ON time with aDBS lies near the lower bound of reported minimal clinically important differences (MCID) for diary‐based outcomes.^97^ Notably, aDBS refines an existing DBS system rather than representing DBS initiation. Although data on ON‐time gains from cDBS reprogramming are limited, clinical experience and observational reports suggest these improvements are typically incremental and of similar magnitude. By contrast, initiation of device‐aided therapies, including DBS implantation, is associated with substantially larger benefits, often approximately 3 to 5 hours/day.^98^, ^99^, ^100^

Improvements in the Movement Disorder Society‐Unified Parkinson's Disease Rating Scale, part III (MDS‐UPDRS III) scores modestly favored DT‐aDBS, consistent with prior observations of modest but reproducible effect sizes.^97^ Total electrical energy delivered (TEED) was reduced by 13% with ST‐aDBS and 11% with DT‐aDBS, with further reductions when patients self‐selected their preferred adaptive settings. Safety outcomes were favorable as most stimulation‐related adverse effects resolved during calibration, and no serious device‐related adverse events were seen during extended follow‐up. Of the 45 participants who entered the long‐term phase, 44 continued aDBS, with 63% reporting improved motor symptoms, 45% fewer side effects, and 66% reduced symptom fluctuations, while quality of life scores remained unchanged.

Only a few studies have explored LFP‐based biomarkers beyond β activity for aDBS.^67^ Oehrn et al^69^ demonstrated the feasibility and clinical benefit of γ‐guided aDBS using stimulation‐entrained γ oscillations (∼60–90 Hz), which tracked dopaminergic state and motor fluctuations more accurately than STN β activity. In a blinded crossover study, personalized γ‐guided aDBS adjusted stimulation according to the dopaminergic state, significantly reducing time with bothersome motor symptoms without worsening others, supporting its potential to improve residual motor fluctuations in PD.

## Gaps and Controversies

### Biomarker

β LFPs, the most common biomarker, show variable prevalence.^14^, ^101^ The ADAPT‐PD trial observed bilaterally elevated α/β activity (8–30 Hz) in 65.2% of patients,^96^, ^102^ whereas other studies reported detection rates of 78% to 95% *off* medication.^102^, ^103^, ^104^ In contrast, a study on intraoperative recordings found bilateral β activity in only 47.25% of 91 patients,^105^ likely reflecting differences in β‐band definitions, inclusion of α frequencies, and recording timing relative to post‐surgical stun effects, which may have reduced—but not entirely suppressed—β expression levels.^56^, ^106^, ^107^, ^108^, ^109^, ^110^, ^111^

Chronic sensing studies show that β amplitude is generally stable over months to years after postoperative stabilization,^112^, ^113^, ^114^ but intra‐ and inter‐day variability and drift with medication necessitate periodic threshold review.^20^, ^96^ Early postoperative suppression of β power can confound contact selection and calibration, supporting deferral of electrophysiology‐guided programming or aDBS activation until approximately 4 weeks post‐surgery or longer if β has not stabilized.^109^, ^110^

Across‐patient analyses indicate that β‐band activity explains approximately 17% to 20% of the variance in motor symptom severity.^44^, ^115^ Although this highlights substantial interindividual variability,^116^ such group‐level associations do not directly reflect the within‐patient, moment‐to‐moment β dynamics that adaptive DBS leverages for real‐time control.

β‐band activity shows limited and inconsistent relationships with non‐motor domains such as mood, cognition, sleep, or autonomic function, and the feasibility of adaptive control for these symptoms remains uncertain. Recent reviews highlight substantial conceptual and technical challenges, underscoring that aDBS for non‐motor symptoms is still exploratory.^117^

Non‐β biomarkers, including γ‐based control signals, remain underexplored.^68^ At‐home recordings show that entrained γ may provide a robust basis for adaptive control,^69^ supporting future longitudinal trials. The relative contribution of factors not directly affecting aDBS—such as interindividual variability in β expression and responsiveness to stimulation—as well as confounders remains poorly understood. β does not index tremor and population‐level correlations with γ are modest (0.11–0.24). DT‐controlled systems, aiming to maintain β within target ranges, face uncertainty regarding optimal control targets. FTG and higher‐frequency biomarkers (>125 Hz) are limited by current device sampling, restricting evaluation mostly to intraoperative settings. Symptom‐specific frequency relationships are inconsistent across patients, as bradykinesia associates with low‐β, tremor with tremor‐frequency, and dyskinesias with θ/γ, yet behavioral states such as sleep and gait modulate these signals in complex ways.^87^, ^90^, ^118^, ^119^

Patient‐reported outcome measures (PROMs) further complicate biomarker use as objective signals sometimes diverge from subjective feedback^120^ Approximately 45% of patients in the ADAPT‐PD trial preferred aDBS despite worse ON‐time without troublesome dyskinesias compared with cDBS.^21^ This highlights a potential mismatch between motor outcomes and subjective experience. Patient preference may reflect benefits not captured by standard metrics, such as perceived stability, reduced unpredictability of fluctuations, fewer side effects at specific times of day, or an increased sense of confidence or control. These observations support the integration of PROMs as complementary signals in the evaluation—and potentially control—of adaptive DBS. This is in line with the significant improvement in general well‐being using EMA questionnaires in a recent aDBS study.^8^ Notably, recent studies indicate patients can reliably guide DBS programming via visual analogue scales.^121^, ^122^, ^123^, ^124^

### Artifact Handling

Artifact handling remains a major challenge, as minimally processed power spectra can be distorted by movement, tremor, and dyskinesias, leading to inappropriate adaptive control. More advanced artifact‐mitigation strategies must balance reliability gains against added hardware, and computational costs, particularly for AI‐based approaches. The value of supplementary inputs (eg, electrocorticography or wearable sensors) for distinguishing true motor symptoms from artifacts and improving generalizability across patients and states remains uncertain.^125^, ^126^, ^127^ Despite these limitations, early movement‐responsive aDBS systems—largely detecting voluntary movement rather than pathological symptoms—represent an important milestone.^128^

### Control Strategies

Control strategies remain an open challenge, including optimal controller–driver pairing and selection of thresholds and polling rates for chronic aDBS. Fixed β thresholds (eg, ±25% around baseline) may inadequately capture symptom dynamics. Despite mechanistic differences, ST and DT approaches show similar clinical outcomes and patient preference, suggesting comparable efficacy. ST may suit rapid events (eg, freezing of gait), whereas DT may better address slower fluctuations and dyskinesias and is often favored in practice because of fewer programming constraints and closer resemblance to cDBS under narrow amplitude windows.

### Clinical Application

The ADAPT‐PD trial suggested that β‐based aDBS is both feasible and safe for chronic use in PD.^21^, ^94^, ^95^, ^96^ Despite these encouraging results, several factors remain to be further defined. Of the originally enrolled 68 participants, 33.8% were excluded from analysis because of intolerance to aDBS or insufficient LFP signal quality, indicating that real‐world applicability may be restricted to a subset of patients with stable β activity. Moreover, only 44% (n = 30) of participants tolerated both evaluated control mechanisms, underscoring the complexity of individualized calibration. Although 63% (n = 43) of participants chose to remain on aDBS for extended follow‐up, those reverting to cDBS were excluded from further participation, potentially biasing tolerability and satisfaction outcomes. ADAPT‐PD revealed substantial interindividual variability in response to aDBS, with superiority over cDBS observed in approximately 45% to 65% of patients depending on control strategy and differential responses between ST and DT modes. This heterogeneity highlights the current lack of reliable predictors for patient or algorithm selection. Importantly, longer‐term follow‐up indicates that most patients continue to use aDBS beyond the trial period, suggesting sustained perceived benefit despite variable short‐term outcomes.

The ADAPT‐PD study provides valuable long‐term evidence supporting the feasibility of personalized aDBS, but also exhibits methodological limitations, as comparisons between aDBS and cDBS were confounded by open‐label assessments, introducing potential expectation and attention biases in diary‐ and clinician‐rated outcomes. Tolerability and safety inferences relied largely on unblinded patient preference, clinician impressions, and selective continuation of participants who tolerated aDBS during setup, raising survivorship and generalizability concerns. The claim of therapeutic equivalence rests on post hoc endpoint adjustments and descriptive rather than confirmatory analyses, while reductions in TEED were nominal and not multiplicity‐controlled across modes. Furthermore, safety evaluation lacked exposure‐adjusted incidence rates and a contemporaneous cDBS comparator, limiting interpretation of “favorable” safety claims.

Selecting appropriate candidates for aDBS remains a major unresolved challenge. The field currently lacks consensus on the threshold of β activity required for reliable application, leaving the definition of “β‐negative” patients uncertain. Although β oscillations comodulate rigidity and bradykinesia, their relationship with tremor is weaker, suggesting that β‐based algorithms may not equally address all motor features of PD.^115^, ^129^ This highlights a critical gap that symptom‐specific biomarkers may be necessary to tailor aDBS more effectively. Tremor, for instance, may involve distinct pathophysiological mechanisms, including lower‐frequency oscillations (θ or α bands) or irregular bursting patterns, which are not captured by β‐based control mechanisms.^119^ Conversely, patients with tremor did not experience worse outcomes in recent trials.^20^, ^21^ This may indicate that β‐based control is most effective in patients with robust β signals or that treatment success is driven less by the specific control algorithm and more by a shared underlying factor.

Robust biomarkers for freezing of gait remain elusive, and real‐time detection is technically challenging given its episodic and multifactorial nature.^130^, ^131^, ^132^ A small pilot study showed that aDBS guided by STN β burst duration was safe and well tolerated, with gait improvements comparable to cDBS and additional benefits for tremor and bradykinesia. However, these preliminary findings require validation in larger controlled studies to identify patients most likely to benefit.

### Practical Considerations

Energy efficiency in β‐based aDBS remains uncertain. Although aDBS is often assumed to reduce battery usage relative to cDBS, data from the ADAPT‐PD trial show only a modest 11% to 13% reduction in energy consumption,^94^ consistent with prior methodological work.^19^ Neural sensing itself increases power demand—by up to approximately 15% even with modest sampling rates^133^—and future aDBS implementations may require faster polling or more complex feature analyses, further increasing energy use. Earlier reports of approximately 40% energy savings likely reflected short‐term, small‐sample laboratory studies, whereas ADAPT‐PD provides more ecologically valid, 30‐day real‐world data. Although battery depletion is less critical for patients with rechargeable Impantable Pulse Generators (IPGs),^134^ energy efficiency remains clinically relevant given its impact on device longevity and replacement surgery risk, the primary source of DBS‐related infections.^133^

**FIG. 3:**
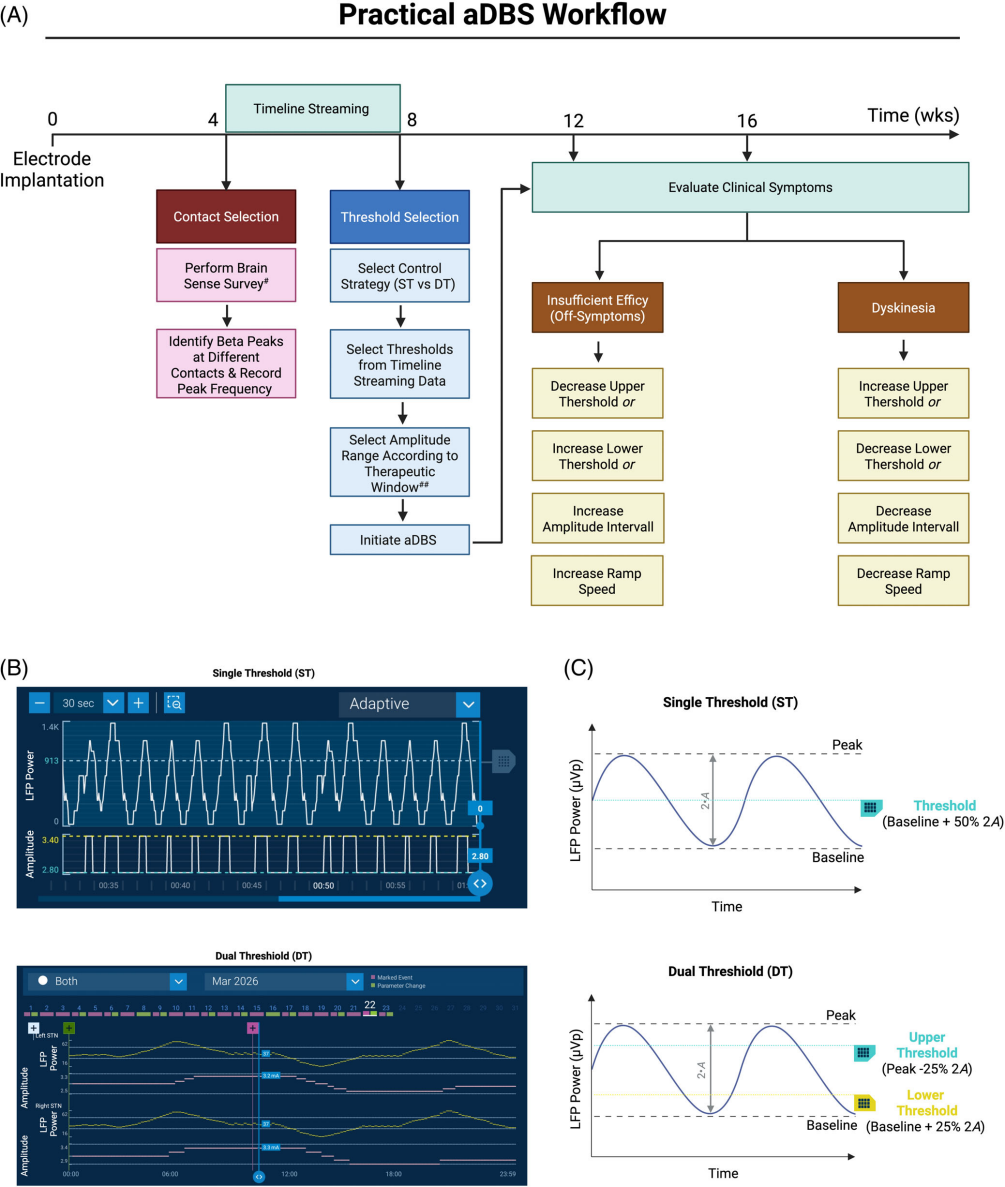
(**A**) Schematic illustrating a practical workflow for adaptive deep brain stimulation (aDBS) setup. ^#^A brainstem survey should be performed in both medication (Med)‐*on* and Med‐*off* conditions. ^##^The therapeutic window is determined clinically by identifying the threshold for clinical efficacy (lower threshold) and the threshold for side effects (upper threshold). (**B**) Representative screenshots illustrating control strategies for single‐threshold (ST) and dual‐threshold (DT) adaptive deep brain stimulation (aDBS). (**C**) Schematic depicting local field potential (LFP) power fluctuations and corresponding threshold settings for ST and DT aDBS. In the ST strategy, the threshold is typically set at baseline plus 50% of the signal range. In the DT strategy, the upper threshold is set at the peak minus 20%, and the lower threshold at baseline plus 20% (signal range: 100%). [Color figure can be viewed at wileyonlinelibrary.com]

Unlike cDBS, aDBS requires an additional calibration phase after postoperative stabilization to confirm stable β activity across medication states and simple motor tasks (Fig. 3A). Programming involves reviewing sensing data, selecting sensing and stimulation contacts, and individualizing control thresholds, often defined as a therapeutic β window in DT paradigms (Fig. 3B,C) or a single control point in ST approaches.^21^, ^94^, ^95^, ^96^, ^135^ Because β dynamics vary with medication, disease progression, and chronic stimulation, thresholds require periodic reassessment based on chronic sensing and clinical response. Compared with cDBS, aDBS shifts programming from repeated manual adjustments toward ongoing supervision of signal validity and control alignment, and patients should be counselled that benefits may require iterative optimization. A step‐by‐step algorithmic approach to aDBS programming and threshold adjustment has been proposed to support clinical implementation.^20^

It remains unclear whether aDBS confers long‐term or neuroplastic effects beyond those of cDBS, which primarily produces acute, stimulation‐dependent benefits.^13^ Although cumulative effects suggesting neuroplasticity have been reported with anterior thalamic DBS for epilepsy,^136^ comparable long‐term modulatory effects of aDBS have yet to be demonstrated.

### Economical and Ethical Aspects

Although closed‐loop DBS requires more initial setup than open‐loop systems, aDBS may ultimately reduce programming time, provided biomarkers are stable and algorithms appropriately selected.^137^ However, large‐scale trials comparing the time‐ and cost‐effectiveness of aDBS versus cDBS are lacking. Beyond economics, aDBS raises ethical concerns, such as potentially higher device costs and unequal clinical resources may exacerbate healthcare disparities; expanding capabilities prompt questions about elective device replacement despite uncertain benefit; continuous neural monitoring introduces privacy and data ownership challenges; and automated stimulation decisions may affect patient autonomy, informed consent, mood, cognition, and perceptions of identity. Uncertainty around patient selection and predictors of benefit complicates fully informed consent, particularly as adaptive algorithms assume greater control over stimulation, underscoring the need for transparency, fallback options, and continued patient involvement. Formal cost‐effectiveness analyses of adaptive DBS are currently lacking. At present, there is no evidence for uniformly higher device costs compared with cDBS, and economic implications related to programming time, clinical workload, and potential reductions in energy consumption remain insufficiently quantified. Consequently, the real‐world economic impact of aDBS cannot yet be reliably assessed and will need to be addressed in prospective health‐economic studies alongside clinical outcome trials.

## Conclusions

Single biomarkers are unlikely to be universally sufficient for aDBS, underscoring the need to integrate multiple signals.^15^, ^41^, ^138^ Machine‐learning models trained offline may further enhance individualized biomarker combinations while remaining computationally efficient in clinical use.^139^ Non‐rhythmic (aperiodic) signal features may also carry clinically relevant information largely neglected by current aDBS.^140^, ^141^ Combining electrophysiological and peripheral measures (eg, electromyography, motion sensors, heart rate variability) should be a research priority to increase responsiveness and personalization without adding excessive system complexity (Fig. 1).

PROMs, such as visual analogue scales, can complement and further personalize neural biomarkers by providing subjective feedback on efficacy and side effects, potentially enabling more granular aDBS control.^20^, ^121^, ^122^, ^123^, ^124^ Emerging AI‐based digital proxies (eg, sleep, voice, and facial metrics) from widely used smart devices may offer additional, continuous inputs. Developing secure digital ecosystems to integrate these multimodal data sources with electrophysiology is a crucial step toward individualized aDBS frameworks, and it offers a compelling use case for conceptualizing brain–AI interfaces.

Integrating electrophysiological and imaging data may enhance aDBS efficacy.^7^, ^120^, ^142^ A theoretical framework termed adaptive circuit‐targeting is envisaged to decode symptom severity from neural signals in real time and to dynamically integrate the activation of brain circuits most relevant to symptom control.^143^

Enhancing artifact rejection improves signal fidelity, but increases energy use and hardware complexity. Empirical studies should define the real‐world prevalence and impact of artifacts to justify these trade‐offs. Implementing hybrid, low‐power denoising techniques (adaptive thresholding, on‐chip filtering, machine learning‐based suppression) could balance performance and efficiency. Standardized testing and benchmarking datasets are recommended to ensure reproducibility and cross‐platform comparability.^33^, ^88^

In clinical practice, selection for adaptive DBS should prioritize the reliability and stability of the neural control signal over motor phenotype. β‐based systems are most readily implemented in bradykinesia‐ and rigidity‐dominant PD, where β activity most consistently reflects motor state and treatment response,^32^, ^37^, ^144^ although tremor‐dominant patients may benefit when a robust biomarker is present.^20^ Patients should not be excluded based on phenotype alone, instead, clinicians should confirm a sufficiently stable β signal using sensing capabilities. As β activity may be transiently suppressed after implantation, aDBS should be initiated no earlier than 1 month postoperatively.^109^ Ongoing evaluation of biomarker stability and adaptive algorithm parameters is essential, and in the absence of a reliable control signal, cDBS should remain the default.

Given limited resources, near‐term aDBS research should prioritize identifying predictors of individual benefit, standardizing biomarker reliability, and developing pragmatic calibration and programming workflows. Subsequent efforts may focus on biomarker validation, state estimation and prediction,^145^ automated tuning, adaptive feedback, and connectome‐informed targeting, supported by registries and comparative trials. Longer‐term goals include multimodal biomarkers, AI‐driven adaptive control, and potential disease‐modifying effects. Because electrophysiological correlates overlap across symptom domains, aDBS is expected to move beyond a single β‐based control signal. Although β modulation effectively targets rigidity and bradykinesia, symptom‐specific or multimodal approaches may better capture quality‐of‐life–relevant outcomes, as reflected by variable PD Questionnaire‐39 improvements.

Clinicians must ensure informed consent given current technological limitations and uncertainties in long‐term outcomes.^20^, ^96^ Moreover, routine use of sensing‐enabled devices will build practical expertise and accelerate safe, cost‐effective adoption. Transparent, interpretable **AI** algorithms and strong data governance frameworks are essential to maintain autonomy, privacy, and fairness. Ongoing health‐economic analyses need to assess cost‐effectiveness and equitable access across healthcare systems.

## Author Roles

(1) Research Project: A. Conception, B. Organization, C. Execution; (2) Statistical Analysis: A. Design, B. Execution, C. Review and Critique; (3) Manuscript: A. Writing of the First Draft, B. Review and Critique.

T.K.: 1A, 1B, 1C, 3A, 3B.

M.S.: 1A, 1B, 1C, 3A, 3B.

A.E.: 1A, 3B.

A.F.: 1A, 3B.

A.A.K.: 1A, 3B.

J.K.K.: 1A, 3B.

## Financial Disclosures for the previous 12 months

T.K. has been supported by the Munich Clinician Scientist Program (MCSP). T.K. has received research funding from Abbott and Medtronic, and honoraria for speaking engagements from Abbott and AbbVie. J.K.K. is a consultant to Boston Scientific and Medtronic and received fees from Aleva and Inomed. A.F. has stock ownership in Inbrain Pharma and has received payments as consultant and/or speaker from AbbVie, Abbott, Acadia, AskBio, Boston Scientific, Ceregate, Dompé Farmaceutici, Inbrain Neuroelectronics, Ipsen, Medtronic, Integrative Research Laboratories Sweden, Iota, Syneos Health, Merz, Sunovion, Paladin Labs, UCB, and Sunovion. He has received research support from AbbVie, Boston Scientific, Medtronic, Praxis, ES and receives royalties from Springer. A.A.K. has received payments as consultant and/or speaker from Boston Scientific, Inbrain Neuroelectronics, Ipsen, and Medtronic. A.A.K. is supported by the Deutsche Forschungsgemeinschaft (DFG, German Research Foundation), Project ID 424778381‐TRR 295. A.E. has received funding from the French Ministry of Education and Research and honoraria for speaking engagements from Medtronic, Boston Scientific, and AbbVie. Author disclosures are available in the Supporting Information.

## Data Availability

Data sharing not applicable to this article as no datasets were generated or analysed during the current study.
